# Human immunodeficiency virus-associated vacuolar encephalomyelopathy with granulomatous-lymphocytic interstitial lung disease improved after antiretroviral therapy: a case report

**DOI:** 10.1186/s12981-020-00295-y

**Published:** 2020-07-09

**Authors:** Kazumasa Akagi, Kazuko Yamamoto, Asuka Umemura, Shotaro Ide, Tatsuro Hirayama, Takahiro Takazono, Yoshifumi Imamura, Taiga Miyazaki, Noriho Sakamoto, Hirokazu Shiraishi, Hideaki Takahata, Yoshiaki Zaizen, Junya Fukuoka, Minoru Morikawa, Kazuto Ashizawa, Katsuji Teruya, Koichi Izumikawa, Hiroshi Mukae

**Affiliations:** 1 Department of Respiratory Medicine, Nagasaki University Hospital, Sakamoto 1-7-1, Nagasaki City, Nagasaki 852-8501 Japan; 2 Infection Control and Education Center, Nagasaki University Hospital, Sakamoto 1-7-1, Nagasaki City, Nagasaki 852-8501 Japan; 3 Department of Neurology and Strokology, Nagasaki University Hospital, Sakamoto 1-7-1, Nagasaki City, Nagasaki 852-8501 Japan; 4 Department of Rehabilitation Medicine, Nagasaki University Hospital, Sakamoto 1-7-1, Nagasaki City, Nagasaki 852-8501 Japan; 5 Department of Pathology, Nagasaki University Hospital, Sakamoto 1-7-1, Nagasaki City, Nagasaki 852-8501 Japan; 6 Department of Radiology, Nagasaki University Hospital, Sakamoto 1-7-1, Nagasaki City, Nagasaki 852-8501 Japan; 7 Department of Clinical Oncology, Nagasaki University Graduate School of Biomedical Sciences, Sakamoto 1-7-1, Nagasaki City, Nagasaki 852-8501 Japan; 8 AIDS Clinical Center, National Center for Global Health and Medicine, Toyama 1-21-1, Shinjuku, Tokyo 162-8655 Japan

**Keywords:** HIV, AIDS, Encephalopathy, Vacuolar myelopathy, Granulomatous-lymphocytic interstitial lung disease, Antiretroviral therapy

## Abstract

**Background:**

Vacuolar encephalomyelopathy, a disregarded diagnosis lately, was a major neurological disease in the terminal stages of human immunodeficiency virus (HIV)-1 infection in the pre-antiretroviral therapy (ART) era. Granulomatous-lymphocytic interstitial lung disease (GLILD) was classically identified as a non-infectious complication of common variable immunodeficiency; however, it is now being recognized in other immunodeficiency disorders. Here, we report the first case of GLILD accompanied by vacuolar encephalomyelopathy in a newly diagnosed HIV-infected man.

**Case presentation:**

A 40-year-old Japanese man presented with chronic dry cough and progressing paraplegia. Radiological examination revealed diffuse pulmonary abnormalities in bilateral lungs, focal demyelinating lesions of the spinal cord, and white matter lesions in the brain. He was diagnosed with GLILD based on marked lymphocytosis detecting in bronchoalveolar lavage, and transbronchial-biopsy proven T-cellular interstitial lung disease with granulomas. Microbiological examinations did not reveal an etiologic agent. The patient was also diagnosed with HIV-associated vacuolar encephalomyelopathy on the basis of an elevated HIV viral load in cerebrospinal fluid. After initiating ART, the brain lesions and paraplegia improved significantly, and interstitial abnormalities of the lungs and cough disappeared.

**Conclusion:**

This report highlights that even in the post-ART era in developed countries with advanced healthcare services, HIV-associated vacuolar encephalomyelopathy should be considered in the differential diagnosis of a progressive neurological disorder during the first visit. Furthermore, GLILD may represent an HIV-associated pulmonary manifestation that can be treated by ART.

## Background

Vacuolar myelopathy (VM) is a syndrome that primarily affects the spinal cord, and predominantly presents in patients with low CD4 cell count. VM presents as a slowly progressing spastic paraparesis with urinary disturbances. In approximately 46.5% of acquired immunodeficiency syndrome (AIDS) cases, VM was identified during the postmortem examination [1], with only 26.8% of these cases experiencing clinical symptoms [2]. In the pre-antiretroviral therapy (ART) era, neurologic dysfunction was recognized as the first manifestation in 10–20% of AIDS patients [3]. VM and encephalopathy were considered fatal, with death occurring within several months after diagnosis [4]. Although HIV-associated neurological diseases in the post-ART era have highlighted neurocognitive impairment, HIV-associated VM with advanced AIDS has rarely been recognized [1].

Patients with AIDS frequently develop lung disease. This is mostly due to opportunistic infections; nonetheless, malignancy and interstitial lung diseases have also been associated with the syndrome. Granulomatous-lymphocytic interstitial lung disease (GLILD) is a diffuse lung disease characterized by both granulomatous and lymphoproliferative histopathologic patterns in the lung [5]. Although GLILD was classically identified as a non-infectious complication of common variable immunodeficiency (CVID), it is now being recognized in other primary immunodeficiency disorders [6]. However, HIV-associated GLILD has not been reported previously. To our knowledge, this is the first report of HIV-associated GLILD and VM occurring simultaneously in a newly diagnosed HIV patient, successfully treated with ART.

## Case presentation

A 40-year-old Japanese man who had a non-productive cough for a year presented with difficulty in walking and pollakiuria, both of which had progressed over a 4 month period. Upon visiting a general practitioner, the patient was referred to a neurologist who hospitalized him for further examination and treatment of spastic paraplegia.

A diagnosis of subacute myelitis of unknown etiology was made, and 1000 mg of methylprednisolone was administered for 3 days; however, the symptoms did not improve. Upon further interrogation, the patient disclosed that he had prior sexual encounters with men. Upon examination tests revealed that he was HIV-1 positive, and he was transferred to our hospital. The patient had no recent travel history and was an office worker. Notably, 10 years prior to his admission, he had been treated for herpes zoster with herpetic keratitis on the left eye. About 6 months prior to his admission, he was receiving mianserin hydrochloride, flunitrazepam, and zolpidem tartrate for depression. He was an ex-smoker with a 30 pack-year smoking history who did not drink alcohol or use illicit drugs.

At admission, the patient had a body temperature of 37.4 °C; heart rate of 103 beats/min; respiratory rate of 16 breaths/min; SpO_2_ of 97% in normal room air; white tongue coating; superficial lymph nodes that were not swollen; and absence of abnormal heart sounds, lung rales, skin, or edema. Examination of the cranial nerves and upper limbs revealed unremarkable results. He manifested spastic gait but no sensory disturbance. The muscle tone had increased, whereas the muscle strength in the lower extremities had weakened, indicating manual muscle testing (MMT) of grade 2. He was unable to rise from the bed on his own. His lower extremity reflexes were brisk bilaterally, and he also had nocturia. Neuropsychological tests conducted by certified neuropsychologists demonstrated normal cognitive function (mini mental state score, 30/30), but revealed attention disorders (trail making test (TMT)-A and -B; 95 and 94 s, respectively) and memory disturbance (Miyake memory test unrelated words score, 5-4-6/10). Eye examination performed by an ophthalmologist revealed mild uveitis in the left eye.

The patient’s blood test results were as follows: leukocytes, 4300/μL (lymphocytes 29.9%); CD4 cell count, 185/μL; HIV-1 viral load, 3.4 × 10^4^ copies/mL; β-d glucan, 35.8 pg/mL (Fungitech G test MK II); Krebs von den Lungen-6, 800 U/mL; and soluble interleukin 2 receptor, 757 U/mL. Serum immunoglobulins were within normal range, and autoantibodies, including rheumatoid factor, antinuclear antibodies, and antineutrophil cytoplasmic antibodies, were all negative. Vitamin B12 and folate levels were normal, and cryptococcosis antigen test, interferon-gamma release assay, anti-mycobacterium avium complex antibody, toxoplasma IgG test, amoebic antibody, and syphilis screening tests were negative. Hepatitis B virus, hepatitis C virus, and human T cell leukemia virus type I, and cytomegalovirus (CMV), were also negative. The results of the cerebrospinal fluid (CSF) analysis were as follows: 16 white blood cells/mm^3^ of which 94% were mononuclear cells; 54 mg/dL glucose; 130 mg/dL protein; and 1.44 IgG index (IgG in CSF, 16.0 mg/dL; albumin in CSF, 24 mg/dL; IgG in serum, 1840 mg/dL; albumin in serum, 3980 mg/dL). CSF viral polymerase chain reaction (PCR) test results were positive for HIV-1 with 4.1 × 10^5^ copies/mL and negative for CMV, herpes simplex virus (HSV), Epstein–Barr virus, varicella zoster virus, John Cunningham virus, BK virus, and Simian vacuolating virus 40. CSF was also negative for cryptococcal antigen testing, and for culture tests for *Mycobacterium tuberculosis* and bacteria. Cytology of CSF was negative.

**Fig. 1:**
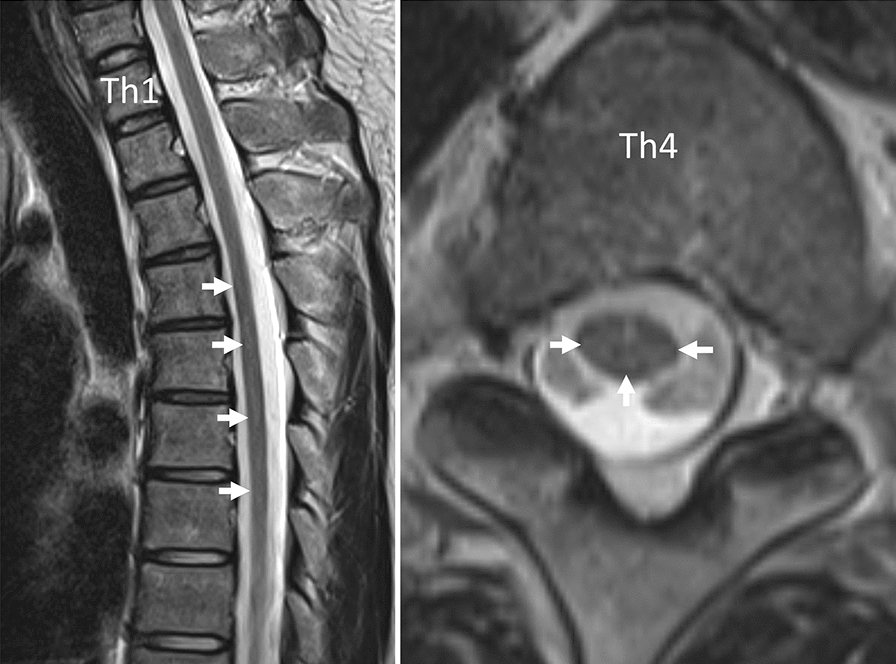
Spine MRI on admission. The sagittal T2-weighed MR image of the spine shows focal demyelinating lesions of the spinal cord from the 4th through the 7th vertebral body (arrowhead), and diffuse spinal atrophy

**Fig. 2:**
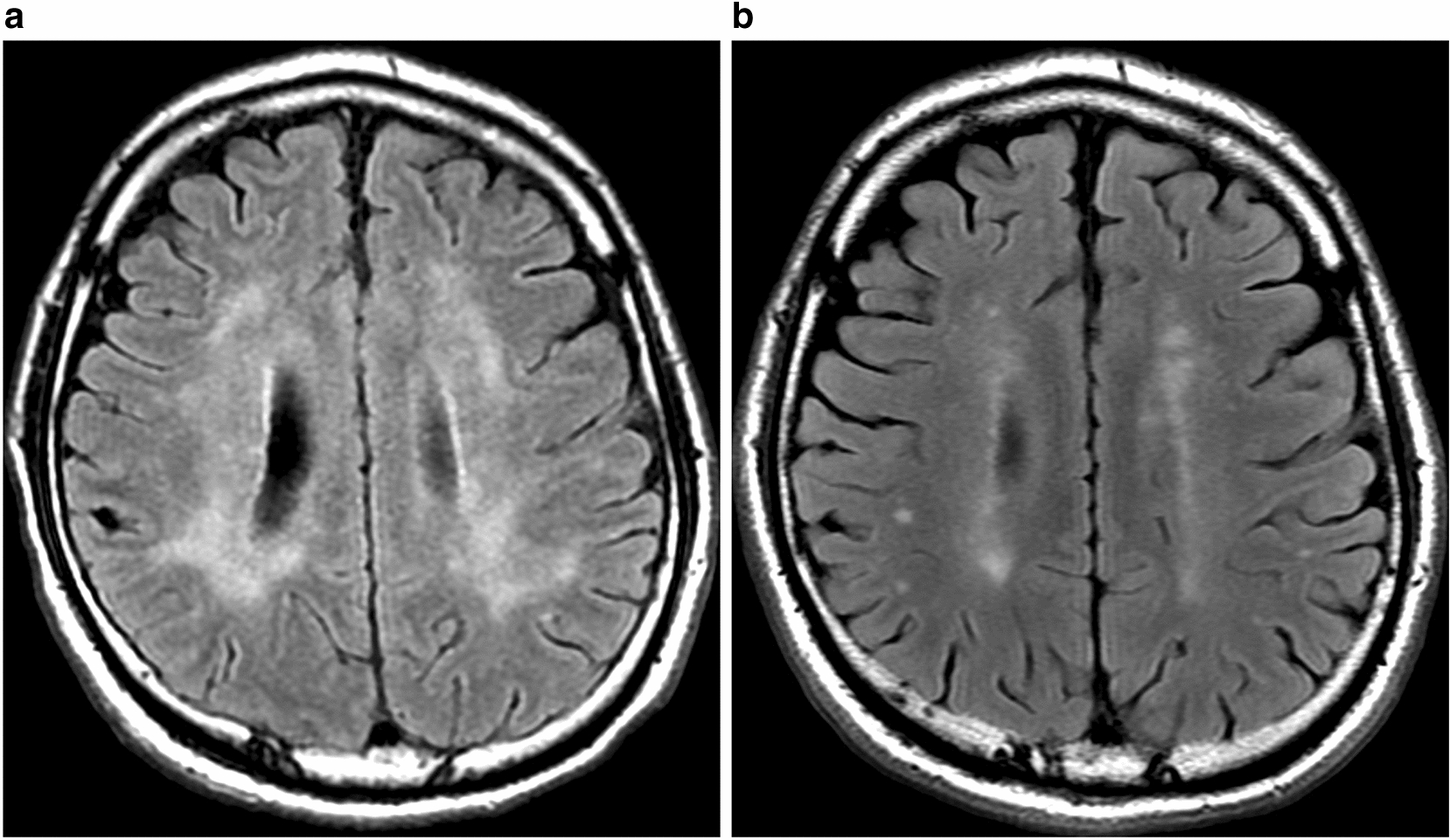
Axial FLAIR brain MR image on admission (**a**) and post-ART (**b**). **a** Symmetrical and diffuse cortical and central atrophies and an extensive high signal of the white matter were detected. **b** A decrease in white matter signals compared to pre-ART

**Fig. 3:**
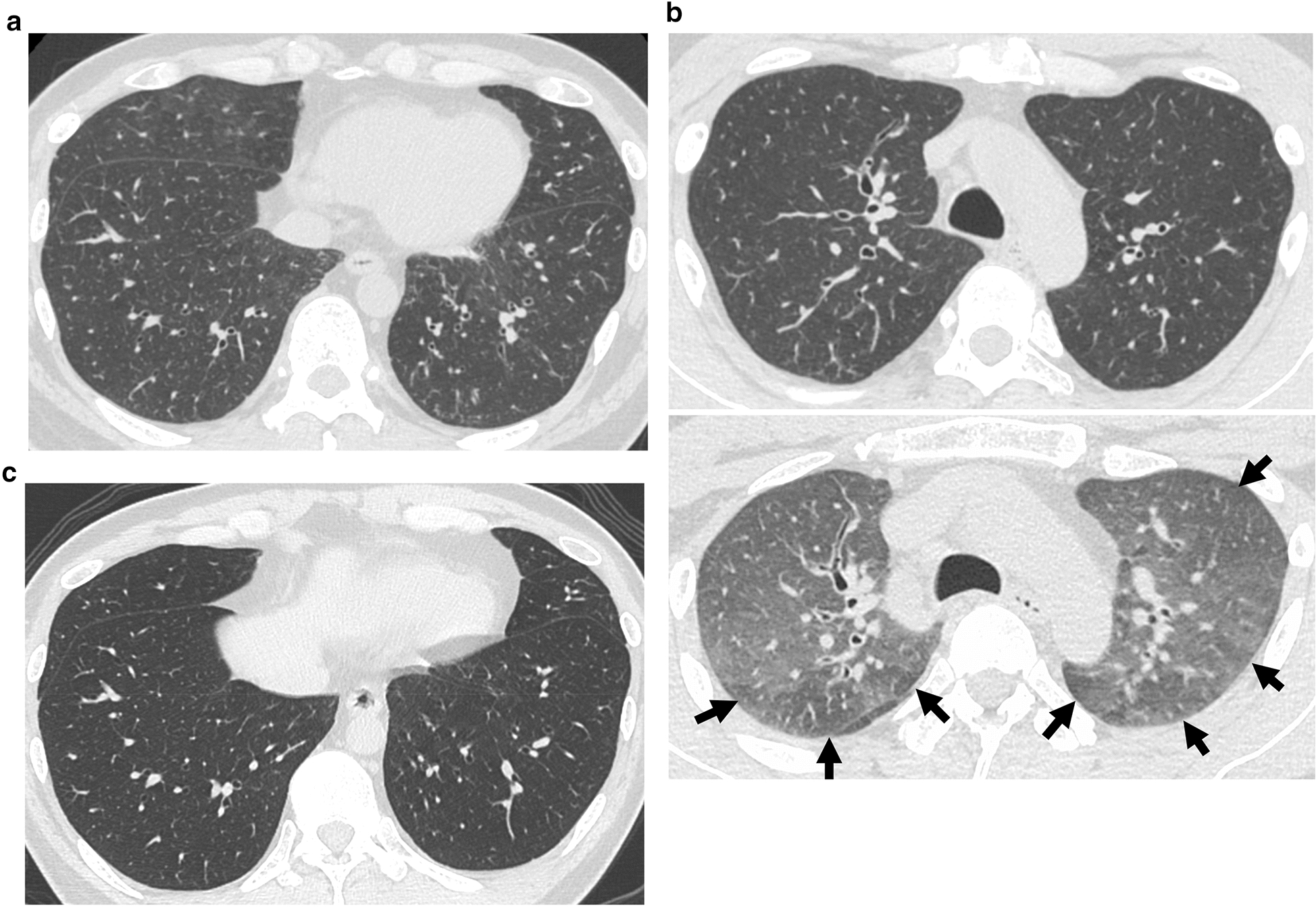
Axial HRCT image of the chest on admission (**a**, **b**) and post-ART (**c**). **a** Multiple centrilobular small nodules and branching opacities within all lung lobes, associated with small areas of ground-glass opacities (GGO) in the peribronchiolar region and bronchial wall thickening are seen. **b** Axial HRCT image on expiratory scan (lower image) shows lobular and subsegmental areas of mosaic pattern (arrowheads) due to air trapping in small airways, which is not evident from the inspiratory scan (upper image). **c** Improvement in interstitial abnormalities of the lung compared to pre-ART

**Fig. 4:**
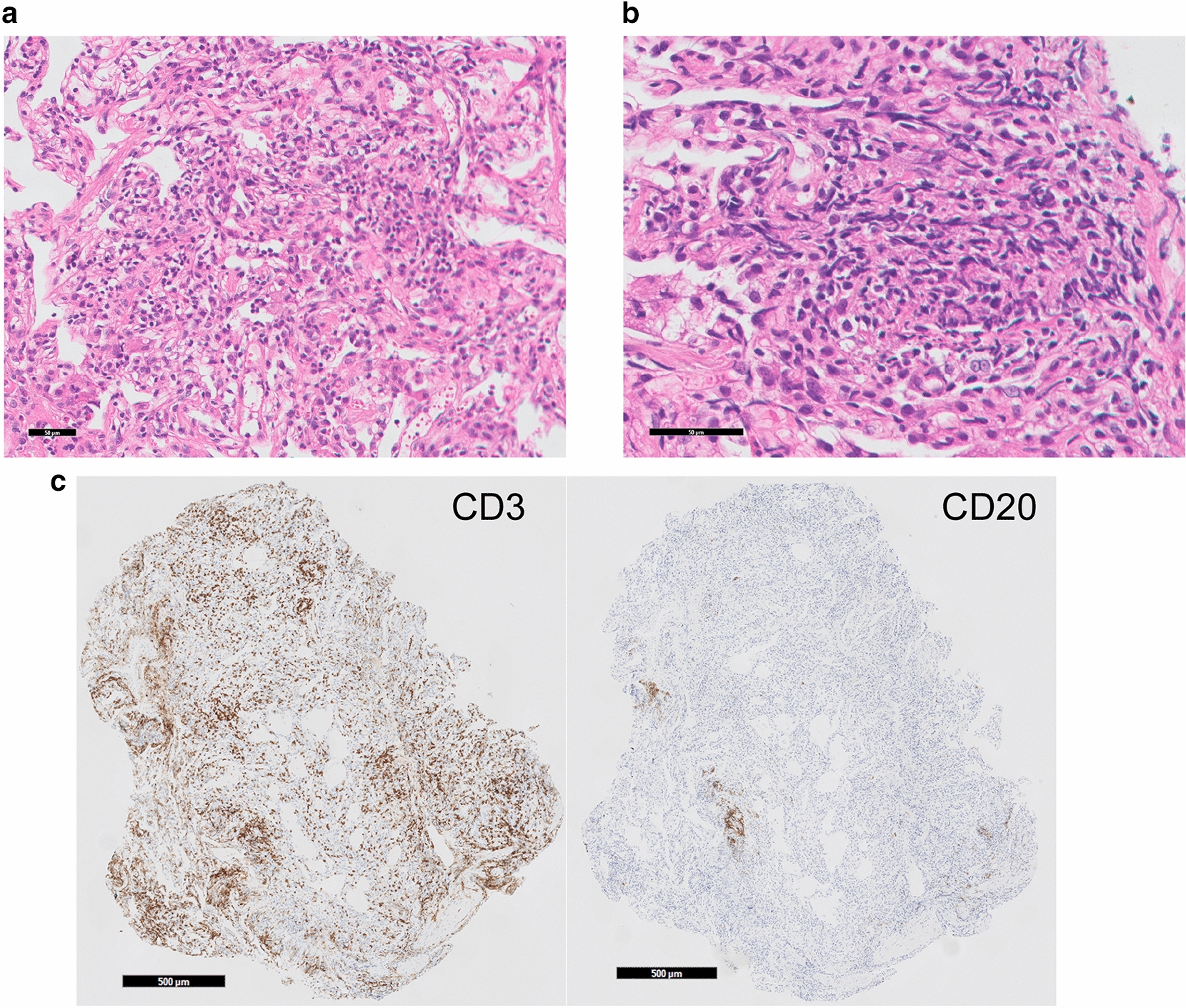
Histopathological features of the TBLB specimen. **a** Marked lymphocyte infiltration without forming lymphoid follicle is identified in the alveolar septa (hematoxylin and eosin [H&E] staining, magnification ×200. Scale bar = 100 micro m). **b** Histiocytes with hinged nuclei are aggregated to form non-necrotizing granuloma (arrow heads, H&E, magnification ×400. Scale bar = 50 micro m). **c** Most of the lung-infiltrated lymphocytes are CD3-positive T cells (left, CD3; right, CD20, both magnification ×40, Scale bar = 500 micro m)

The T2-weighed transverse spinal MRI (Fig. 1) showed focal demyelinating lesions of the spinal cord from the 4th through the 7th vertebral body, and diffuse atrophies of the spinal cord. Axial FLAIR MR images of the brain (Fig. 2a) showed confluent white matter hyperintensity and diffuse brain atrophies, whereas no brain mass lesions were detected. Chest X-ray indicated bilateral reticulonodular opacities predominantly in the lower lung zones. High-resolution computed tomography (HRCT) of the chest showed multiple centrilobular small nodules and branching opacities within all lung lobes, which were associated with small areas of ground-glass opacity (GGO) in the peribronchiolar region and bronchial wall thickening (Fig. 3a). A mosaic pattern was noted on expiratory HRCT image (Fig. 3b) showing air-trapping in the small airways. Mild mediastinal lymphadenopathies were also observed. A pulmonary function test revealed vital capacity of 4.25 L (103.7%), forced expiratory volume in 1.0 s/forced vital capacity of 78.9%, and decreased diffuse capacity for carbon monoxide of 57.9%. Bronchoalveolar lavage (BAL), and transbronchial lung biopsy (TBLB) were conducted on day 10 of admission. The number of cells in the BAL fluid (BALF) was 5.0 × 10^5^/mL, with a cell differentiation of alveolar macrophages (18.5%), neutrophils (8.4%), lymphocytes (46.6%), and eosinophils (26.5%). The CD4/8 ratio of BALF was 0.05; cultures for bacteria and mycobacteria including tuberculosis were negative; and PCR test results using BALF for HSV, CMV, and *Pneumocystis jirovecii* were all negative. Hematoxylin and eosin staining of the TBLB specimen showed marked lymphoid infiltrate in the alveolar septa (Fig. 4a). Occasional non-necrotizing granulomas composed of epithelioid histiocytes were found in the lung field (Fig. 4b). Among lung-infiltrating lymphocytes, T cells were dominant (Fig. 4b) with a relatively higher number of CD8 cells than CD4 cells. Neither dense fibrosis nor microorganisms were found on Elastin van Gieson, Grocott, or Ziehl–Neelsen staining. There were no CMV inclusion bodies or toxoplasma cysts in either the TBLB lung specimen or BALF cytology. Based on these findings, the patient was diagnosed with HIV-associated vacuolar encephalomyelopathy and HIV-associated GLILD.

Treatment with emtricitabine/tenofovir alafenamide fumarate and dolutegravir and prophylaxis with 1 g of trimethoprim-sulfamethoxazole (TMP-SMX) per day was initiated on day 9 of admission. An indwelling catheter was inserted due to neurogenic bladder dysfunction. One week after ART introduction, muscle weakness in the lower extremities and bladder disturbance temporarily worsened but improved after 3 weeks. The MMT of the lower extremities recovered from grade 2 to 4, and the patient was able to walk with a walker. The indwelling catheter was removed, and intermittent self-catheterization was started on day 30 of hospitalization. Blood test results 2 months after initiating ART revealed that the CD4 count had increased to 296/μL, HIV viral load decreased to 26 copies/mL, and β-d glucan decreased to a normal level. HIV-RNA was undetectable 4 months after initiating ART. The patient was discharged after 84 days of hospitalization, ambulating with a cane and his dry cough had disappeared. Brain MRI 50 days after initiating ART showed a marked decrease in white matter lesions (Fig. 2b), and spinal MRI showed no interval change compared with that pre-ART. HRCT of the chest at 2 months after initiating ART showed improvement in interstitial abnormalities of the lung (Fig. 3c). The neuropsychological test performed after the discharge demonstrated that the patient’s attention disorders had improved (TMT-A and -B were 41 and 60 s, respectively).

## Discussion and conclusion

VM typically has a slow progression over several months and causes weakness in the lower extremities without causing sensory disturbance [7]. VM is a diagnosis of exclusion, and other spinal cord diseases (infectious and non-infectious) that may mimic this condition must be ruled out. Vitamin B12 deficiency occurs in 13–50% of HIV infected patients and may cause spinal degeneration [8] [9]. In VM, spine MRI or CSF studies rarely show abnormalities [7]. The diagnosis of VM in our patient was supported by focal demyelinating lesions in the spinal cord on T2-weighed MRI [10], normal serum Vitamin B12 level, and the CSF findings of protein elevation, lymphocytic pleocytosis [7], and HIV replication [11]. Negative PCR results of JC virus in CSF cannot exclude progressive multifocal leukoencephalopathy (PML) [12]; however, the clinical course and MRI findings in our case were atypical for PML. Apart from initiation of ART in antiretroviral-naïve patients [13–15], there are no proven effective treatment options for HIV-associated VM [16], and, in fact, our case did not respond to steroid treatment. Whether VM responds to ART in a similar way as other primary neurological manifestations of HIV infection is controversial [17]. Our case highlights the benefits of ART in HIV-associated vacuolar encephalomyelopathy, with improvement in clinical symptoms and MRI abnormalities.

GLILD in adults is defined as ‘‘a distinct clinico-radio-pathological interstitial lung disease occurring in patients with CVID, associated with a lymphocytic infiltrate and/or granuloma in the lung, and in whom other conditions have been considered and where possible excluded [5] ’’. While this consensus definition focuses on patients with CVID, we suggest its extrapolation to include other immunodeficiencies in which GLILD is becoming increasingly recognized [6], such as in the case of our patient diagnosed with AIDS. The etiology of GLILD is yet undetected, but decreased CD4 T cells might be related to the development of GLILD [6]. Respiratory symptoms are often nonspecific with pulmonary function tests indicating a mild restrictive disorder with a more marked decrease in diffusing capacity [6], as seen in our case. Radiographically, diffuse pulmonary nodules with a lower predominance and surrounded by a GGO is predominant in GLILD [6]. Histologically, evidence of granulomatous inflammation with lymphoproliferative changes, including lymphoid interstitial pneumonia (LIP), follicular bronchiolitis (FB), and/or diffuse reactive lymphoid hyperplasia with T cell predominance as typically seen in GLILD [5]. In our case, diffuse mixed interstitial lung and small airway disease were seen in HRCT, lymphocytosis in the lung was revealed by BAL, and granulomatous T-cell-dominant lymphoproliferative inflammation mainly in the interstitial and peri-bronchial lesions were found by TBLB. The differential diagnoses of GLILD included infections such as miliary tuberculosis, sarcoidosis, and lymphoma [5]. The CD4 count < 200/µL and a high β-d glucan level in our patient pointed to *Pneumocystis jirovecii* pneumonia (PCP). However, as the CT findings indicated a lack of cystic lesions and a negative PCR result of BALF, PCP was unlikely [18]. In addition, a prophylaxis dose of TMP-SMX is usually insufficient to treat PCP. A possible mechanism of GLILD has been proposed by impaired T-cell function, which leads to dysfunctional antigen handling, and/or aberrant responses to viral infection [5]. Prognosis of GLILD varies, due to a combination of factors, including the natural history of the disease, existing comorbidities, and treatment response [6]. Although the effectiveness of ART treatment for HIV-associated GLILD was not reported, LIP [19] and/or FB [20] in adult HIV patients can be resolved by ART.

Our case report demonstrates that even in the post-ART era in developed countries with advanced healthcare services, HIV-associated vacuolar encephalomyelopathy should be considered as a differential diagnosis of a progressive neurological disorder. In addition, this case highlights the importance of a comprehensive work-up in case of diffuse pulmonary abnormalities in HIV patients, including BAL and lung biopsy, and to exclusion of infectious and neoplastic diseases to reach a definitive diagnosis.

## Data Availability

Data sharing is not applicable to this article as no datasets were generated or analyzed during the current study.

## Abbreviations

VM: Vacuolar myelopathy
AIDS: Acquired immunodeficiency syndrome
ART: Antiretroviral therapy
GLILD: Granulomatous-lymphocytic interstitial lung disease
MMT: Manual muscle testing
CMV: Cytomegalovirus
CSF: Cerebrospinal fluid
PCR: Polymerase chain reaction
HSV: Herpes simplex virus
HRCT: High-resolution computed tomography
BAL: Bronchoalveolar lavage
BALF: BAL fluid
TBLB: Transbronchial lung biopsy
TMP-SMX: Trimethoprim-sulfamethoxazole
PCP: *Pneumocystis jirovecii* pneumonia
